# Dilation of Malignant Strictures in Endoscopic Ultrasound Staging of Esophageal Cancer and Metastatic Spread of Disease

**DOI:** 10.1155/2011/356538

**Published:** 2011-11-30

**Authors:** Shawn M. Hancock, Deepak V. Gopal, Terrence J. Frick, Patrick R. Pfau

**Affiliations:** Division of Gastroenterology and Hepatology, Department of Medicine, University of Wisconsin School of Medicine and Public Health, 4241 MFCB, 1685 Highland Avenue, Madison, WI 53705, USA

## Abstract

*Background*. Dilation of malignant strictures in endoscopic ultrasound (EUS) staging of esophageal cancer is safe, but no data exists regarding the subsequent development of metastases. *Aim*. Compare the rates of metastases in esophageal cancer patients undergoing EUS staging who require esophageal dilation in order to pass the echoendoscope versus those who do not. *Methods*. We reviewed consecutive patients referred for EUS staging of esophageal cancer. We evaluated whether dilation was necessary in order to pass the echoendoscope, and for the subsequent development of metastases after EUS at various time intervals. *Results*. Among all patients with similar stage (locally advanced disease, defined as T3, N0, M0 or T1-3, N1, M0), there was no difference between the dilated and nondilated groups in the rates of metastases at 3 months (14% versus 10%), *P* = 1.0, 6 months (28% versus 20%), *P* = 0.69, 12 months (43% versus 40%), *P* = 1.0, or ever during a mean followup of 15 months (71% versus 55%), *P* = 0.48. *Conclusions*. Dilation of malignant strictures for EUS staging of esophageal cancer does not appear to lead to higher rates of distant metastases.

## 1. Introduction

Endoscopic ultrasound (EUS) is an important part of staging for esophageal cancer. It provides key information regarding local tumor invasion, locoregional, and celiac lymph node involvement. This information is essential to guide future treatment decisions [1–9]. Often a malignant stricture is present that prohibits passage of the echoendoscope for complete EUS staging. Earlier studies showed that dilating these malignant strictures led to high complication rates [1, 10]. More recently, the safety of dilating malignant strictures for passage of an echoendoscope for esophageal cancer staging has been well established [2–4, 11]. Safety data in previous studies primarily focused on rates of perforation. Currently there are no data available on whether dilating malignant strictures may precipitate metastatic spread of cancer.

At our institution, it was noted by our thoracic surgery and oncology section that there were a high number of distant metastases in unusual locations shortly after surgery in patients who had been dilated at the time of pretreatment EUS staging. This led to a request by the thoracic surgery section to limit dilation for the performance of EUS in the staging of esophageal cancer patients.

Numerous reports of iatrogenic periprocedural spread of cancer cells in many other procedures exist, including seeding needle tracts in breast biopsies [12–14], diagnostic and therapeutic procedures for hepatocellular carcinoma [15–17], cutaneous seeding in laparoscopic cholecystectomy [18–21], and seeding tracts with fine needle aspiration (FNA) in pancreatic, esophageal, and thyroid lesions [22–26]. It is also well known that dilation of esophageal strictures carries a high rate of transient bacteremia [27–30], presumably through the breakdown of tissue planes and bacteria seeding the bloodstream. In theory, a similar mechanism could occur with cancer cells seeding the bloodstream during dilation of a malignant stricture, but this has not been previously documented or noted. The goal of our study is to ascertain if dilating malignant strictures in EUS esophageal cancer staging lead to higher rates of metastases.

## 2. Methods

Our institution uses a multimodality staging regimen for esophageal cancer including EUS, CT scan, positron emission tomography (PET) scan, or combined CT-PET scan. There was no predetermined order of the various staging tests. We reviewed 55 consecutive patients referred for EUS for the purpose of staging esophageal cancer. All patients had biopsy-proven esophageal cancer. EUS was performed by 3 endoscopic ultrasonographers (D. V. Gopal, T. J. Frick, and P. R. Pfau) with experience ranging from 5 to 10 years with an average of 200 EUS exams per year per endoscopist, using an Olympus GF-UM130 or GF-UM160 radial array echoendoscope (Olympus America, Melville, NY, USA) with both 7.5 and 12.0 mHz frequencies. Malignant strictures were only dilated if the stricture prevented passage of the echoendoscope. Dilation was performed sequentially with either wire-guided dilation or a through-the-scope balloon. No strictures were dilated beyond 15 mm. EUS staging was done immediately after dilation using the American Joint Committee on Cancer (AJCC) Tumor, Node, Metastasis (TNM) staging system, 6th edition.

Patients' electronic medical records were reviewed to obtain all data. We collected data on the patients age, gender, histology of cancer, location of malignant stricture (cervical esophagus, thoracic esophagus, or gastroesophageal junction) if dilation was required to pass the echoendoscope, TNM stage at the time of EUS, and whether or not distant metastases had been identified at certain time intervals—0 (the time of original EUS staging), 1, 3, 6, and 12 months following EUS, or at any time beyond 12 months if applicable. Survival data was also collected using the Social Security Death Index. Patients who had evidence of distant metastases on the pretreatment staging were not included in the analysis. Ascertainment of the presence of distant metastases at the chosen time intervals was done by reviewing all imaging studies and clinic notes through the given time interval on each patient. This study was approved by the University of Wisconsin Health Sciences Institutional Review Board.

Two groups of patients were compared, those who required dilation in order to pass the echoendoscope and those who did not. Patients with locally advanced disease (defined as T3, N0, M0 or T1-3, N1, M0) were identified in both the dilated and nondilated group and compared with each other in order to attempt to match the patients in the two groups for similar stage at the time of EUS.

Statistical analysis comparing the dilated group and nondilated group was performed with Chi-square test or Fisher's exact test where appropriate.

## 3. Results

55 consecutive patients were identified. 23 patients required dilation in order to pass the echoendoscope, 32 did not. The echoendoscope was successfully passed through the malignant stricture following dilation allowing full staging in 21 of the 23 patients in the dilated group. There was no difference between the two groups with respect to age, sex, location of stricture, or histology of cancer (Table 1). There were no procedure-related complications in either group.

15 of the 55 patients had distant metastases present at the time of EUS, 9 in the dilated group and 6 in the nondilated group (*P* = 0.13). In these 15 patients, EUS was done on the same day or within the same week as the CT or PET that detected the distant metastases. The remaining 40 patients had no distant metastases at the time of the pretreatment staging EUS. Of these 40 remaining patients, 14 required dilation at the time of the staging EUS and 26 did not undergo dilation at the time of staging EUS. These 40 patients formed the basis for the study's comparison.

**Figure 1:**
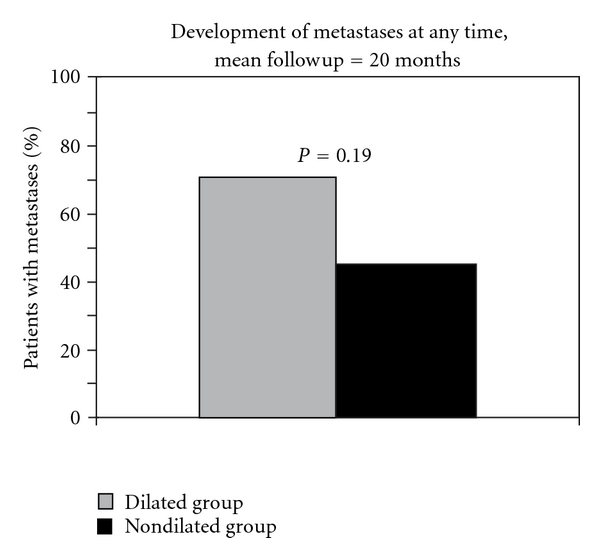
Development of metastases at any time during a mean followup of 20 months among all patients without metastases present at the time of staging EUS.

Of these 40 patients who had no evidence of metastatic disease at the time of original staging, 10 of 14 (71%) in the dilated group and 11 of 26 (46%) in the nondilated group went on to develop metastases at any point during a mean followup of 20 months (range 1–125) (*P* = 0.19) (Figure 1). Metastases were detected at a median of 10 months (range 1–54) after EUS in the dilated group and 10 months after EUS in the nondilated group (range 1–125). Metastases were identified in a variety of locations in both the nondilated group and the dilated group (Table 2). 

**Figure 2:**
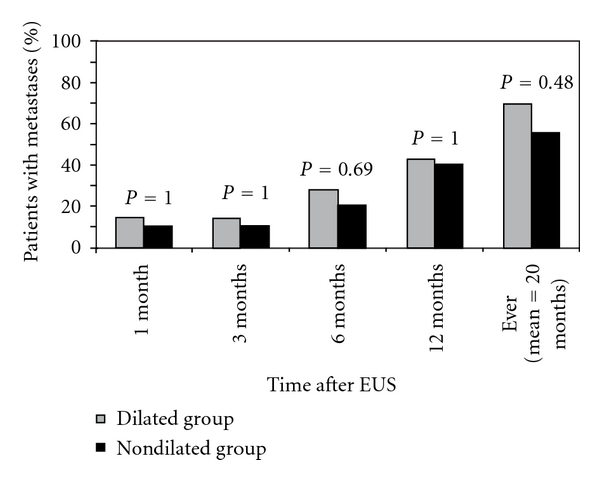
Development of metastases at various time intervals after EUS among patients with similar staging-locally advanced disease.

Patients from the dilated and nondilated groups were further compared to account for similar stage at the time of EUS. All 14 patients in the dilated group and 20 out of 26 patients in the nondilated group had locally advanced disease (defined as T3, N0, M0 or T1-3, N1, M0). Excluding all patients with distant metastases at initial staging, the exact dilated group stages were as follows: T3N1 (*N* = 8), T2N1 (*N* = 1), and T3N0 (*N* = 5); the exact nondilated group stages were as follows: T3N1 (*N* = 8), T2N1 (*N* = 5), T1N1 (*N* = 1), T3N0 (*N* = 6), T2N0 (*N* = 1), and T1N0 (*N* = 5). Among these patients with locally advanced disease, the results were as follows: at both 1 and 3 months after EUS, 2 of 14 (14%) in the dilated group had metastases present compared to 2 of 20 (10%) in the nondilated group (*P* = 1.0); at 6 months after EUS, 4 of 14 (28%) in the dilated group and 4 of 20 (20%) in the nondilated group had metastases present (*P* = 0.69); at 12 months after EUS, 6 of 14 (43%) in the dilated group and 8 of 20 (40%) in the nondilated group had metastases present (*P* = 1.0); 10 of 14 (71%) in the dilated group versus 11 of 20 (55%) in the nondilated group went on to develop metastases at any time during a mean followup of 20 months (*P* = 0.48) (Figure 2). 

 Five-year survival data among all patients, regardless of initial stage, reveal that 1 of 22 patients (4%) in the dilated group was alive at 5-years, compared to 6 of 32 (19%) in the nondilated group (*P* = 0.22). When those with metastases present at initial staging are excluded, the 5-year survival data is 1 of 14 (7%) in the dilated group, compared to 6 of 26 (23%) in the nondilated group (*P* = 0.39). Among those who had locally advanced disease, the 5-year survival rate is 1 of 14 (7%) in the dilated group, compared to 1 of 20 (5%) in the nondilated group (*P* = 1.0).

## 4. Discussion

EUS is an essential part of a comprehensive staging workup for esophageal cancer and, when used appropriately in conjunction with CT and/or PET scan, is generally considered the most accurate tool for staging [31]. Further, one of the most important parts of EUS in staging esophageal cancer is to ascertain the presence of celiac node involvement [32]. The echoendoscope needs to be able to pass the malignant stricture in order to provide celiac node assessment. The need for dilating a malignant stricture in order to pass the echoendoscope is reported to be 10–38% [1–4, 7, 10, 11]. This emphasizes the importance of dilation in completing accurate and comprehensive EUS staging that includes celiac node assessment. Without dilation, these are patients that would have suboptimal staging, which in turn can negatively affect treatment decisions.

Early studies concluded that dilating malignant strictures for the purposes of EUS staging of esophageal cancer was dangerous, leading to unacceptably high rates of perforation [1, 10]. More recent studies have refuted this and confirmed that dilating with smaller diameter Savary or through-the-scope dilators, one can safely dilate malignant esophageal strictures in order to complete EUS staging [2–4, 11].

These previous safety studies focused on perforation rates in dilating malignant esophageal strictures. No studies to date report any relationship between dilating malignant strictures and the subsequent development of metastases. It is well established that dilating esophageal strictures carries a relatively high rate of transient bacteremia [27–30], presumably through the breakdown of tissue planes allowing direct seeding of bacteria into the blood stream. One could postulate that a similar mechanism could lead to cancer cells seeding the bloodstream during dilation of a malignant stricture, but there are no data to support this theory. This theory was considered at our institution because of the observation of the rapid development of metastases in unusual locations for a number of patients who had dilation of their malignant strictures for EUS staging as part of their otherwise negative pretreatment staging. Besides the analogy to bacteremia during dilation as stated above, the possibility of malignant spread may also be supported by reports of iatrogenic periprocedural spread of tumor cells being well described in other procedures, including seeding needle tracts in breast biopsies [12–14], diagnostic and therapeutic procedures for hepatocellular carcinoma [15–17], cutaneous seeding in laparoscopic cholecystectomy [18–21], and seeding tracts with FNA in pancreatic, esophageal, and thyroid lesions [22–26].

Malignant cells have been noted in the blood stream after various invasive procedures. Prostate cells, both benign and malignant, have been isolated in the circulation following transrectal prostate biopsy and transurethral resection of the prostate [33]. Furthermore, iatrogenic periprocedural spread of tumor cells directly into the bloodstream has been documented following percutaneous ethanol injection and transarterial chemoembolization in primary liver cancer [34].

We did find that among all patients (not matched for similar stage at the time of EUS), those who required dilation had a trend towards an overall higher rate of metastases at any time during followup (71% versus 46%) (Figure 1). However, this is not completely unexpected as those patients with higher-grade strictures who required dilation generally have more advanced disease [1, 2, 7, 8, 10], and therefore the need for dilation may be a marker for more advanced or aggressive disease. Thus, these patients may be more likely to develop metastases at an earlier time regardless of dilation.

 Because of this observation, we evaluated patients in the dilated and nondilated groups to match those with similar staging—locally advanced disease. When these groups were analyzed, we saw no difference in the rates of metastases at any time interval studied (Figure 2). Furthermore, there is no distinct pattern of metastatic spread unique to either group, and no distinct pattern of location of metastases based on the time frame they were detected. There is also no difference in the median time to detection of metastases in either group (Table 2).

Survival data show a nonsignificant trend towards lower survival in the dilated group when we look at all patients regardless of initial staging. A similar nonsignificant trend is also seen among those without distant metastases at initial staging. However, when the dilated and nondilated groups were matched for similar initial staging, locally advanced disease, five-year survival rates were similar.

The possibility remains that dilation results in seeding the bloodstream and metastatic spread of disease in a small number of cases. However, this theory has not been proven in any previous model or study in this particular clinical setting. While limited by the total number of patients, our study appears to refute that dilation of malignant strictures leads to increased rates of metastases. A more likely explanation is simply that the cases of early metastases in each group represent metastases that were present but not detectable at the time of initial staging.

Our study was limited by a small sample size, retrospective nature of the study, and being a single-center experience. The small sample size makes it difficult to draw any definitive conclusions; however, our study provides valuable information about the natural progression of metastases in patients who undergo EUS for staging of esophageal cancer.

EUS provides the most accurate locoregional staging for esophageal cancer, and dilation may often be necessary to complete EUS staging. Dilating malignant strictures in order to complete EUS staging does not clearly lead to a higher rate of metastases.

## Figures and Tables

**Table 1:** Demographics of patients with esophageal cancer who did and did not undergo dilation at the time of staging EUS.

	Dilated group	Nondilated group	*P* value
Total patients	23	32	
Men	18	29	0.26
Women	5	3	0.26
Mean age	63	64	
Adenocarcinoma	16	27	0.21
Squamous cell carcinoma	7	5	0.21
Cervical esophagus stricture	2	2	0.99
Thoracic esophagus stricture	6	10	0.77
Gastroesophageal junction stricture	15	20	1.0
Metastases present at time of EUS	9	6	0.13
Locally advanced disease at time of EUS	14	20	1.0

**Table 2:** Location of metastases by time from staging EUS in patients who did and did not undergo dilation at the time of staging EUS.

	Dilated group	Nondilated group
Location of metastases detected at <6 months (total number of occurrences)	*4 patients * Mediastinal lymph nodes (2) Lungs (1) Pleural effusion (1) Axillary lymph nodes (1) Adrenal gland (1) Gastric lymph nodes (1)	*4 patients * Liver (2) Peritoneum (2) Back (1)
Location of metastases detected at 6–12 months (total number of occurrences)	*2 patients * Recurrence at GE junction (2) Diffuse bony metastases (1) Mediastinal lymph nodes (1)	*5 patients * Liver (3) Lungs (2) Pleural effusion (2) Peritoneum (1) Recurrence at GE junction (1)
Location of metastases detected at >12 months (total number of occurrences)	*4 patients * Recurrence at GE junction (1) Axillary lymph nodes (1) Cervical lymph nodes (1) Neck (1) Mediastinum (1)	*3 patients * Recurrence at GE junction (1) Lungs (1) Abdominal mass (1)
Median time to detection of metastases (range)	10 months (1–54 months)	10 months (1–125 months)
